# Ecological factors affecting minerals and nutritional quality of “*Dryopteris filix-mas* (L.) Schott”: an underutilized wild leafy vegetable in rural communities

**DOI:** 10.3389/fnut.2024.1276307

**Published:** 2024-02-20

**Authors:** Nasrullah Khan, Rafi Ullah, Mohammad K. Okla, Mostafa A. Abdel-Maksoud, Ibrahim A. Saleh, Hashem A. Abu-Harirah, Tareq Nayef AlRamadneh, Hamada AbdElgawad

**Affiliations:** ^1^Department of Botany, University of Malakand, Chakdara, Khyber Pakhtunkhwa, Pakistan; ^2^Department of Botany, Dr. Khan Shaheed Govt: Degree College Kabal Swat, Swat, Khyber Pakhtunkhwa, Pakistan; ^3^Department of Botany and Microbiology, College of Science, King Saud University, Riyadh, Saudi Arabia; ^4^Department of Medical Laboratory Sciences, Faculty of Allied Medical Sciences, Zarqa University, Zarqa, Jordan; ^5^Integrated Molecular Plant Physiology Research, Department of Biology, University of Antwerp, Antwerp, Belgium

**Keywords:** *Dryopteris* *filix-mas*, nutritional profile, commercial cultivation, marginal lands, ecological drivers, Hindukush-Himalaya

## Abstract

*Dryopteris filix-mas* (hereafter *D. filix-mas*), a wild leafy vegetable, has gained popularity among high mountain residents in the Hindukush-Himalaya region due to its exceptional nutritional profile, and their commercial cultivation also offers viable income alternatives. Nevertheless, besides phytochemicals with medicinal applications, ecological factors strongly affect their mineral contents and nutritional composition. Despite this, little has been known about how this wild fern, growing in heterogeneous ecological habitats with varying soil physiochemical properties and coexisting species, produces fronds with optimal mineral and nutritional properties. Given its nutritional and commercial significance, we investigated how geospatial, topographic, soil physiochemical characteristics and coexisting plants influence this widely consumed fern’s mineral and nutrient content. We collected soil, unripe fern fronds, and associated vegetation from 27 *D. filix-mas* populations in Swat, NW Pakistan, and were analyzed conjointly with cluster analysis and ordination. We found that the fronds from sandy-loam soils at middle elevation zones exhibited higher nitrogen contents (9.17%), followed by crude fibers (8.62%) and fats (8.09%). In contrast, juvenile fronds from the lower and high elevation zones had lower moisture (1.26%) and ash (1.59%) contents, along with fewer micronutrients such as calcium (0.14–0.16%), magnesium (0.18–0.21%), potassium (0.72–0.81%), and zinc (12% mg/kg). Our findings indicated the fern preference for middle elevation zones with high organic matter and acidic to neutral soil (pH ≥ 6.99) for retaining higher nutritional contents. Key environmental factors emerged from RDA analysis, including elevation (*r* = −0.42), aspect (*r* = 0.52), P-3 (*r* = 0.38), K+ (*r* = 0.41), EC (*r* = 0.42), available water (*r* = −0.42), and field capacity (*r* = −0.36), significantly impacting fern frond’s mineral accumulation and nutrient quality enhancement. Furthermore, coexisting plant species (*r* = 0.36) alongside *D. filix-mas* played a pivotal role in improving its mineral and nutritional quality. These findings shed light on the nutritional potential of *D. filix-mas*, which could help address malnutrition amidst future scarcity induced by changing climates. However, the prevalent environmental factors highlighted must be considered if the goal is to cultivate this fern on marginal lands for commercial exploitation with high mineral and nutrient yields in Hindukush-Himalaya.

## 1 Introduction

Pteridophytes are prehistoric plant species with origins tracing back to the late Devonian era (1), comprising vascular ferns and fern allies that propagate and distribute through spores, distinguishing them from flowering plants that rely on flowers or seeds for reproduction (2). Since the ferns and their allies do not constitute a monophyletic group, therefore, the taxonomic arrangement currently acknowledges the combined grouping of Lycopodiophyta, or fern companions, and Pteridophyta, the true ferns, under the broader category termed Pteridophyta (3, 4). Lycopodiophyta comprised only five relict taxa, i.e., Lycopodium, Isoetes, Selaginella, Phylloglossum, and Stilites (4). The Pteridophyta (ferns) and Lycopodiophyta (club mosses) are conserved vascular plants having parallel lineage along with Bryophytes (mosses) (5). Furthermore, Ferns are vascular plants that produce spores and undergo an alternation of generations. In this regard, Lycopods and ferns are similar, but ferns are the evolutionary more similar to seeded plants (gymnosperms and angiosperms), whereas the Lycopods are sister to all other vascular plants (6).

The diversity of Pteridophyta is far more varied than the Lycopodiophyta in terms of morphological form variation and global distribution (3). These small groups encompass a broad spectrum of species (12,000); notably, several are harvested from their natural habitats for medicinal purposes (2). In contrast, certain members of these groups are cultivated for food and ornamental purposes (7). People in rural areas in developing countries have a tradition of gathering wild edible plants (8), such as in the western Sahel area of sub-Saharan Africa as well as communities in Asia, rely extensively on a variety of wild edible plants, and this dependence rises more under drought circumstances (9, 10). These indigenous tribes in sub-Saharan Africa and Asia use plants gathered from their natural habitats to enhance their diets. This practice primarily revolves around cultivating rain-dependent staple crops such as cassava, maize, millet, sorghum, and wheat. The wide array of wild edible plants contributes to the diversification of diets among rural communities (8), as wild food resources play a crucial role during periods of food scarcity (11). As a result, including wild foods holds significant importance in the livelihood and survival strategies of rural mountainous communities.

**Figure 1:**
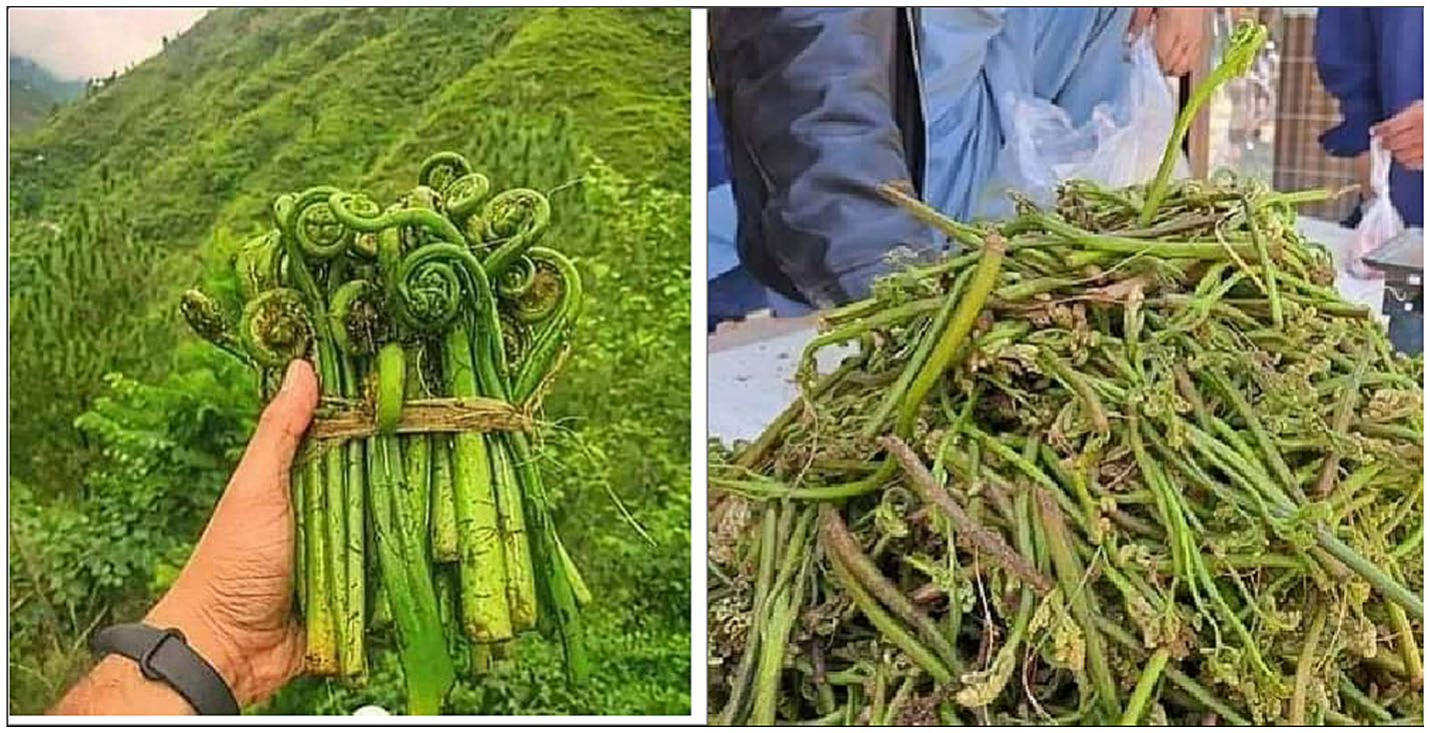
*Dryopteris filix-mas* collected in wild habitat (right) and sold in the market (left).

Among the most popular wild food plants that harvested worldwide are edible ferns (12), which encompass a range of parts, including food-grade fern stems, rhizomes, leaves, immature fronds, shoots, and even entire plants are utilized (13). Pteridophytes, which include ferns, have experienced intermittent attention, notably in regions such as China, Hawaii, Japan, and Nigeria (14). These ferns are traditionally used for treating various ailment since ancient times (15). Herbal remedies may be taken internally in the form of variously prepared concoctions or applied externally as topical applications including lotions, frictions, poultices, drops for the eye, fumigations, baths, and mouthwashes (16). However, the specific utilization of ferns for culinary purposes in Pakistan has received comparatively less research focus (17). An example of particular interest is *Dryopteris filix-mas* (Figure 1), a robust perennial fern with an ornamental value that exhibits homosporous traits and is known for producing hermaphrodite, asexual, male, and female gametophytes (18) holding a remarkable place in traditional cuisine as a wild vegetable (19). Taxonomically, it belongs to the Dryopteridaceae family (12) and is commonly referred to as “wild fern” or “bear’s paw” demonstrates a remarkable adaptability to various habitats, including open ground, most often found in damp areas and on deciduous woods.

This fern extends its natural range across Europe, temperate Asia, North India, North and South America, and the temperate regions within the United States, with occurrence even reaching the African continent (20). The species has extensively been reported to be threatened in the region of United States and United Kingdom, facing various anthropogenic pressures (21). However, due to its remarkable adaptability, this plant can flourish in various soil conditions, from fertile to dry (18). Nevertheless, it prefers environments with moisture and shade, particularly within the forest understory, and tends to thrive in shaded regions such as hedge banks and rocky landscapes (22). The ferns species has various ecological and evolutionary role is community dynamics, i.e., increasing species diversity, forming the forest ground flora, changing the community dynamics and structure, maintain forest community structure and stability (23). These species also have important role in increasing species richness and enhance soil organic matter increasing soil fertility (24). However, due to fragmentation of habitat by human settlement and agricultural lands formation, the fern species has faces sever threat of population disturbance and its dynamics alteration (25). Moreover, these effects are found to be more pronounce in tropical and sub-tropical areas (26). Despite the threats, the young fresh fern fronds are collected and sold as vegetables in the local markets due to their natural abundance in the study area, making them an ideal focal species for research purposes.

Various ecological factors can influence the properties and availability of plant nutrients crucial for plant growth and development (27). In this sense, spatial and soil conditions serve as the immediate living environments of plants, providing the necessary climate, water, and nutrients (28). Therefore, changes in plant nutritional characteristics may be primarily driven by soil nutrient variables. However, the precise interaction between soil and plant nutrients remains enigmatic, especially in intricate regulatory feedback loops between soil and plant nutrients (29). Nutrient-rich soils tend to exhibit more substantial nutrient release upon litter decomposition to sustain greater soil fertility levels. In contrast, nutrient-poor soils generate less plant litter, leading to slower breakdown and soil infertility (30). Additionally, soil attributes such as soil texture and physiochemical parameters like pH and electrical conductivity play a pivotal role in determining concentrations of plant nutrients and can exert either positive or negative effects on nutrient levels in different plant parts (31).

In traditional cuisines, the people of District Swat have long been adept at incorporating diverse wild vegetables. These wild vegetables are consumed in various forms―cooked, raw, or as crisp additions to salads―employing multiple cooking methods. Some plant species produce leaves and flowers that are carefully snipped and then fried for consumption as food. Not just restricted to leaves, rhizomes, stolons, leaf petioles, corms, and sometimes whole plants are eaten. Although these plants are primarily grown for their vegetables, they also have certain therapeutic and nutritive qualities (32). Therefore, the current study aims to evaluate the nutritional value of *Dryopteris filix-mas* and the interplay between environmental and soil variables affecting its nutritional composition. Furthermore, exploring the nexus between fern frond nutrients and ecological factors is undertaken, shedding light on the potential effects of species coexistence ecological and soil variables. The study primarily hypothesized that the nutritional contents of *Dryopteris filix-mas* is related with complex nexus of ecological and environmental variables.

## 2 Materials and methods

### 2.1 Study area

**Figure 2:**
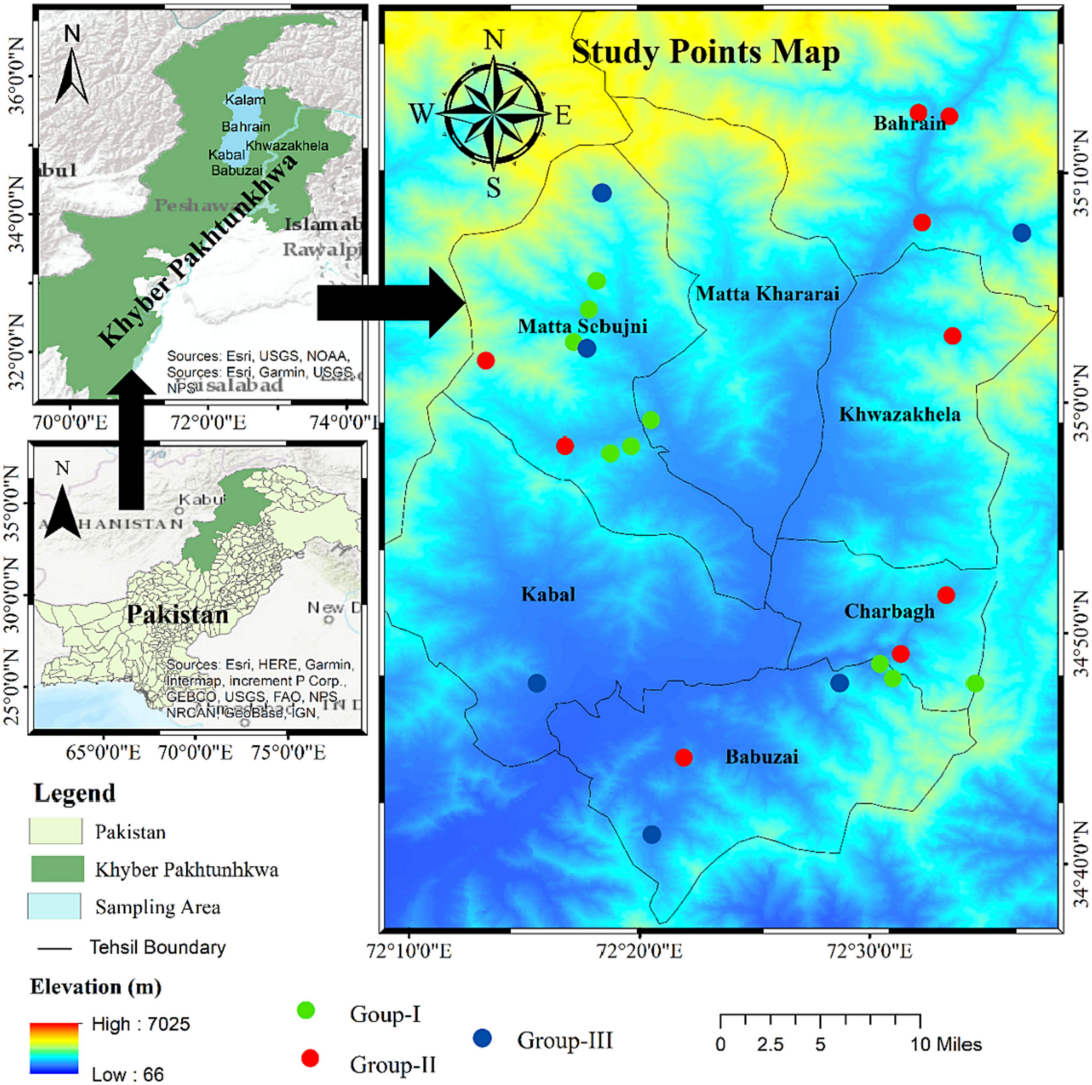
Map of the study area showing sampling sites of *Dryopteris felix-mass* with different color billiards across the elevation.

The sampling sites for this research were chosen within the Swat Hindukush Range of northern Pakistan, in a climatic region characterized by dry and moist temperate forests (Figure 2). These selected sites of *D. filix-mass* populations are distributed across the Sino-Japanese phytogeographical zone of Pakistan above *Pinus roxburghii* and mixed Oak vegetation extended to both dry and humid temperate evergreen coniferous forests (33). The district covers an area of 5,337 km^2^ and lies between latitude 35.2227 N and 72.4258 E longitudes (34), with geological features such as schistose and hornblenditic rocks with distinct felsic rock layers (35). In the low-elevation areas, alluvial soils predominate, providing fertile ground for cultivating cereal crops, fruit, and vegetables (36). The climate of the study area falls under the humid subtropical category with four distinct seasons closely intertwined with elevation gradient, according to the Köppen climate classification (Cfa). High-altitude regions are known for their frigid winters and substantial snowfall, leading to the formation of glaciers, while the summers are short and mild (37). The research area experiences temperatures ranging from 13.4 to 21.7°C and an annual precipitation range of 693–897 mm (38). Notably, November and April are the driest and wettest months, respectively, with precipitation totals of 15 and 93 mm at higher elevations. The dichotomy between the region’s wettest and driest months reveals an average precipitation difference of 112 mm. The region’s warmest month is July (24.1°C on average), while its lowest is January, with an average temperature of 1.5°C (39).

### 2.2 Proximate analysis

In the research region, 30 sample locations were chosen for collecting plant species and proximate analysis. Five plant samples were taken from each site, and the associated species were counted. The associated species were identified using the Pakistani flora to calculate the species diversity indices.

Following Maan et al. (40), three important diversity indices, i.e., Species richness (S), Shannon-Wiener diversity index (H′), and evenness index (E)—were computed based on species density. The number of species in the stand was counted to determine the species richness. The following formulas are used to determine the H′ and E indices Equations 1, 2:


(1) $$H^{\prime }=\overset{S}{\underset{i=1}{\sum }}\mathrm{pi}\;\mathrm{In}\mathrm{pi}$$

(2) $$E=\frac{H^{\prime }}{\mathrm{In}S}$$

Where (*i*) represents the total number of species, *pi* = proportion of the species, S 0 species richness, and In = natural logarithm of *pi*.

#### 2.2.1 Samples preparation

The five wild vegetable samples were washed before air drying in the shade. Samples were ground into powder using a grinder after being air dried, then kept in glass vials for further chemical analysis.

#### 2.2.2 Moisture contents determination

The samples were dried overnight in an oven at 105°C to assess the moisture content. In a petri dish, a 2 g sample was collected and weighed (taken as W1) and again weighed after cooling (taken as W2) (41). The proportion of moisture was calculated using Equation 3.


(3) $$\mathrm{Moisture}\;\mathrm{contents}\%=\frac{W1-W2}{WS}\times 100$$

Note: W1 (Wet sample and Petri dish weight); W2 (Dry sample and Petri weight); and WS (Weight of the sample).

#### 2.2.3 Fat contents determination

The crude fat contents were determined by taking 1 g of moisture-free biomass in a cellulose cartridge (part of Soxhlet’s apparatus). One hundred milligrams of petroleum spirits were used throughout the 6-h extraction process, which took place at 40–60°C. The solvents were evaporated using a rotary evaporator to remove the solvent and extract the crude fats. The flask was given time to cool so that the weight was accurate. The weight difference was estimated using the formula below as a percentage of crude fat (19, 42) using Equation 4.


(4) $$\mathrm{Crude}\;\mathrm{Fat}\%=\frac{W1-W2}{WS}\times 100$$

Note: W1 (Flask weight with fat); W2 (Weight of empty flask); and WS (Weight of the sample).

#### 2.2.4 Ash contents determination

Ash contents % was measured using an empty oven-dried crucible that was cooled in a desiccator before being weighed. The starting weight (W1) was a 1 g sample put into the crucible, weighed, labeled, and burnt on a stove with low heat. The crucible was then placed in a muffle furnace after being burned. The temperature of the furnace was intended to increase gradually from 550 to 660°C. After burning in the furnace for 6 h, the samples took on a grayish-whitish shade. After the burning stage, the sample was removed from the furnace and cooled in a desiccator. As the final weight (W2), the weight was again measured and recorded (42, 43) using Equation 5.


(5) $$\mathrm{Ash}\;\mathrm{contents}\%=\frac{W3-W1}{WS}\times 100$$

W3 (Ash weight); W1 (Initial weight); WS (Weight of the sample).

#### 2.2.5 Nitrogen content determination

The crude protein content of wild plants was assessed using a Kjeldahl apparatus, to which 1 g of sample was added for digestion (42). A digestion mixture (K_2_SO_4_; CuSO_4_) and concentrated H_2_SO_4_ were added to the digestion flask. The mixture was fully mixed by turning the flask, avoiding the development of crystals. The digesting process started when heated in a fume hood and continued until the mixture became clear (blue-green). After digestion, the heating was turned off, and the flask was refrigerated. A Kjeldahl device was used to distill the material that had been digested. The digested sample and 10 mL of sodium hydroxide (NaOH 40%) were added to the distillation tube. NH_3_ was produced and collected as ammonium hydroxide (NH_4_OH) in the receiving flask. The flask was filled with 20 mL of 4% boric acid (H_3_BO_4_) solution and a few drops of methyl red dye. The NH_4_OH progressively changed the pink tint to yellowish. The distillate was titrated against 0.05 N HCl when the pink hue emerged during distillation. In addition, following the previous actions, a blank was executed. The following equation was used to determine the amount of protein using Equation 6:


(6) $$\mathrm{Nitrogen}\%=\frac{\left(S-B\right)\times 0.01\times 100}{WS\times V}\;\times 100$$

Note: S (Titration Sample); B (Blank reading); N (Normality); D (Sample dilution after digestion) V (Volume for distillation); 0.014 (Milliequivalent weight of nitrogen).

#### 2.2.6 Determination of crude fiber

The crude fiber content was assessed using the acid and alkali digestion method (42, 44). 2 g of the sample and 200 mL of 2% HCl were added to a beaker. It was stirred and cooked in a steam bath for 30 min before filtering through a cotton cloth. The same method applied 2% NaOH solution to the residue. The residue was added to the crucible after it had been weighed. The crucible was then put in the desiccator to begin the procedure. The crucible was weighed after cooling and placed in a 550°C furnace for 4 h. After cooling in the desiccator, the crucible was weighed once again. The fibers’ contents were calculated using Equation 7:


(7) $$\mathrm{Crude}\;\mathrm{Fibers}\%=\frac{\begin{array}{l} \mathrm{Weight}\;\mathrm{of}\;\mathrm{the}\;\mathrm{dried}\;\mathrm{residue}\\ -\mathrm{Weight}\;\mathrm{of}\;\mathrm{oven} \end{array}}{\mathrm{dried}\;\mathrm{weight}\;\mathrm{of}\;\mathrm{sample}}\times 100$$

#### 2.2.7 Mineral determination

1 g sample was taken in a flask; 12 mL of nitric acid was added to help digestion, and left the sample to sit for the night. The solution was heated on hot plates, adding 5 mL of perchloric acid until it became transparent. After allowing the samples to cool, Whatman filter paper No. 42 was used to filter them. A volumetric flask was used to hold the filtrate. The solution was examined for several factors using an Atoms Adsorption spectrometer (Shimadzu AA/670) and suitable hole cathode lamps. Applying the general calibration curves produced from the general AR-grade solutions, various mineral nutrients, i.e., K^+^, Mg^++^, Zn^++^, and Ca^++^, were determined (45).

### 2.3 Assessment of soil

The soil characteristics were assessed by auger-bored 3 kg of dirt from two opposing corners and a mid-position of each studied site. To reduce variability, the samples were taken using traditional pedological methods at 0–30 cm depth (46), as the top soil layer is rich in nutrients (47). The samples collected were bulked and thoroughly mixed (40, 47). A pH meter was used in the field to measure the pH of the soil in a soil-water solution (9, 33), and an EC meter was used to measure the electrical conductivity. The physicochemical and textural quality of the soil was evaluated by air-drying the samples and screening them through a 2-mm sieve following USDA standards (48). The Walkley-Black method was used to estimate organic matter, and wet combustion with chromic acid digestion followed by dry combustion was used to assess total and organic carbon (49). Micro-Kjeldahl apparatus was used to determine accessible phosphorus (P^2+^) and exchangeable potassium (K^+^) to assess total nitrogen (50). According to Arend et al. (51), the geometric technique was utilized to calculate lime (calcium carbonate; %) and the geometric method to evaluate CO_2_ evolution. Using an online calculator (52), we also assessed the bulk density (BD), wilting and saturation point (WSP), field capacity (FC), available water (AW), and conductivity (s/cm) of the soil. At the Swat Agriculture Research Institute (ARI), the hydrometer approach evaluated the distribution of silt, clay, and other textural characteristics (53).

### 2.4 Statistical analysis

The bulk of site data and its variation were simplified by classifying it into meaningful groups using Ward’s agglomerative cluster analysis. The variations among plant nutrient concentrations and related environmental factors were assessed using one-way ANOVA, followed by Tukey’s HSD means comparisons. The Pearson’s co-efficient of correlation were used to relate the relationship among the various environmental variables effecting the distribution of nutrient contents in *Dryopteris filix-mas.* The significance threshold of *p* < 0.05 was used to determine the significant change. The principal component analysis (PCA) was used to identify spatial and soil variable and diversity indices with loading and eigenvector values to identify the major statistically different components among the samples. Redundancy analysis (RDA) was used to investigate the nutrient contents of plants and their relation to topography, soil physiochemical, and diversity indices. The RDA analysis seems suitable due to the higher axis gradient, i.e., more than 4 in Detrended correspondence analysis and low variance and axis anarchy in canonical correspondence analysis (38). PC-Ord version 6.0 and the statistical package for social sciences (SPSS) were used to analyze variance, PCA, and RDA. Graph pad version 8 was used to show the nutrient variations in different groups by drawing box plots with mean points of the data.

## 3 Results

### 3.1 Nutrient variation in young fern fronds

**Figure 3:**
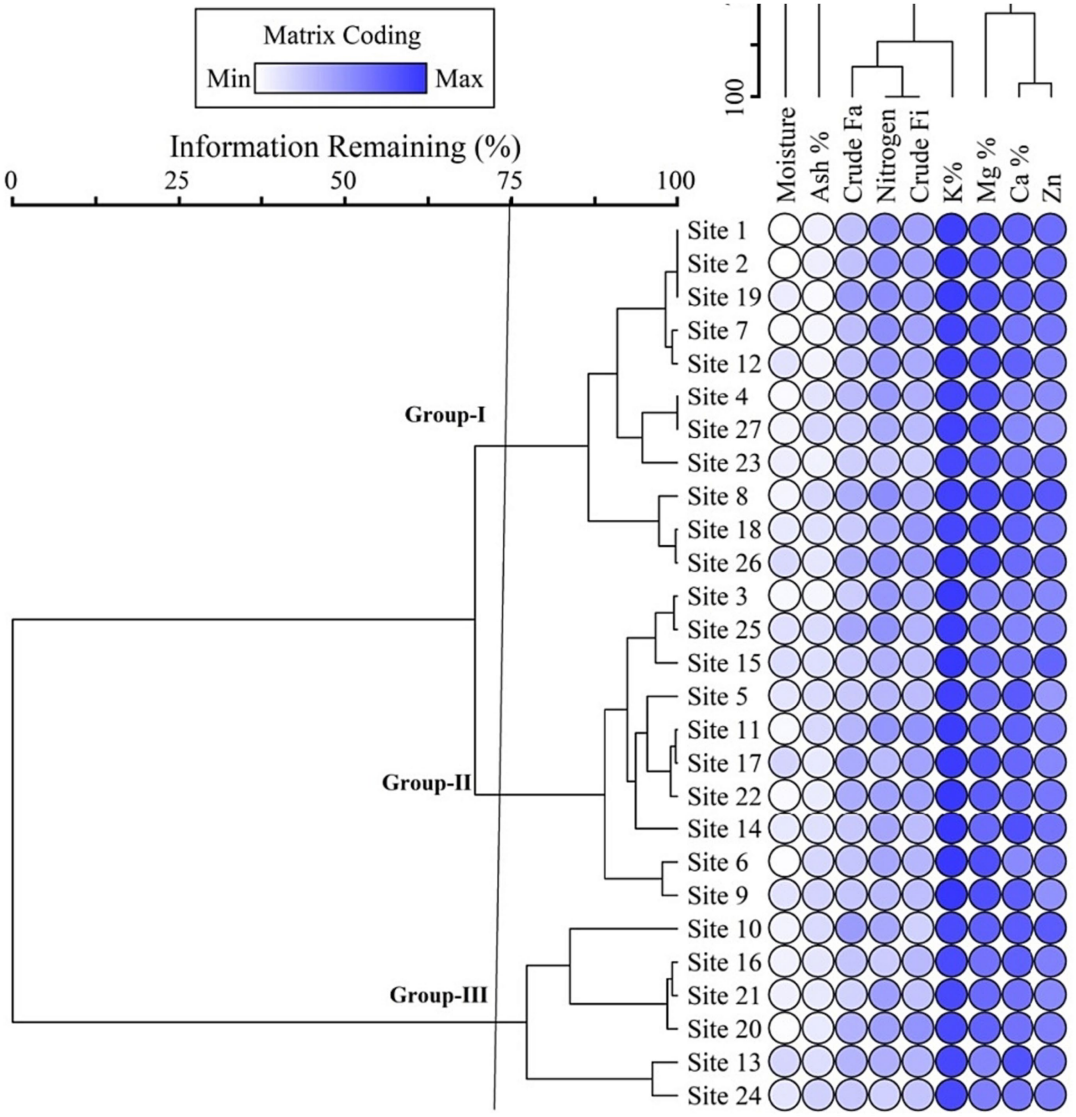
Nutrient-based grouping of stands dominated by *Filix mass* in the study area.

Twenty-seven populations of *D. filix-mas* were categorized into three distinct clusters based on differences in minerals and nutrient composition (Figure 3). The young fronds collected from the largest population, designated as Cluster-I, encompassed 11 sites (Site 1–Site 26) exhibiting higher nitrogen (9.17%), magnesium (0.21%), and potassium (0.81%) contents. Cluster-II, the second largest group (site 3–Site 9), comprised 10 sites characterized by higher moisture (1.98%), crude Fats (8.09%), Fiber (8.62%), and calcium (0.16%) quantities in the juvenile fronds of *D*. *flix-mas* populations. Contrarily, Cluster-III (St 10–Site 24) embodies six populations with lower levels of micronutrients, such as calcium (0.16%) and potassium (0.72%), while displaying the highest quantity of Zn and ash content among all the populations. Likewise, in the proximate analysis, juvenile fronds of *D. filix-mas* exhibited lower amounts of moisture (1.66%), crude fat (5.83%), and fibers (6.61%). The differences in mineral content and proximate analysis among the three populations (Clusters-I–III) are depicted in Figure 3. Notably, the fern fronds sampled from the three *D. filix-mas* populations showed significant differences in crude fat (*F*-value = 17.02; *p* < 0.01) and ash content (*F*-value = 4.72; *p* < 0.05) contents. The nitrogen content in the fronds also displayed considerable variation (*F*-value = 13.47; *p* < 0.001) among the populations, ranging from 7.14 to 9.17% (see Figure 3). Additionally, there was statistically significant variability in crude fiber (6.61–8.62%, *F*-value = 14.01, *p* < 0.001) among the proximate components, while magnesium (Mg^+2^), phosphorus (P^−3^), potassium (K^+^), and carbon (C^+4^) concentrations in the fern fronds did not yield noteworthy variations (Figure 3).

**Figure 4:**
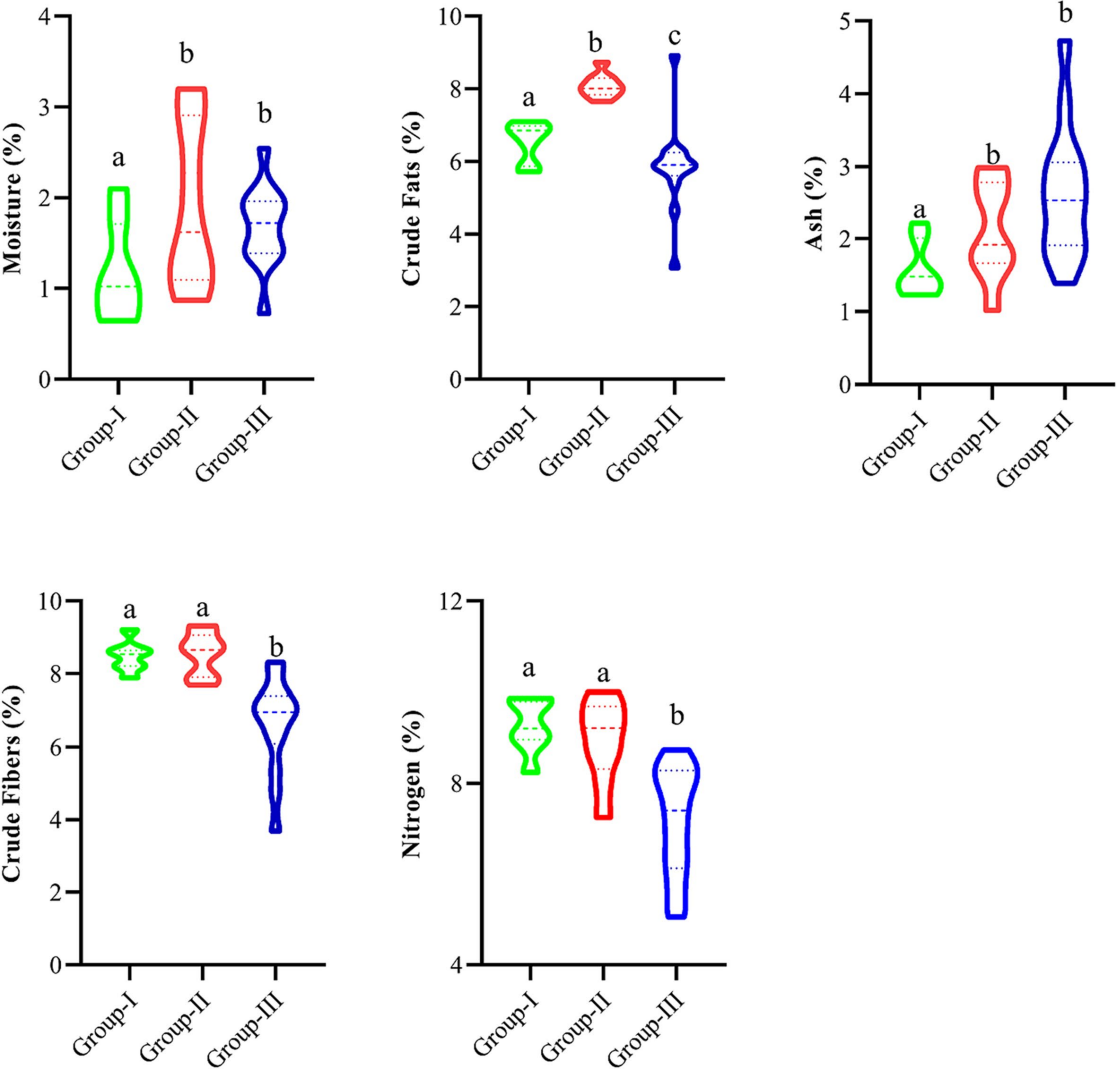
Nutrient descriptive statistics in different groups separated by Ward’s Cluster. Different letters indicate significant differences at *p* < 0.05.

Figure 4 presents *D. filix-mas* proximate value descriptive statics and variation across the groups coupled with elevation and nutrient concentrations of soil. The groups-II was rich in contents of moister percentage (1.98 ± 0.334), crude fat percentage (8.097 ± 0.122), and crude fibers and Carbon percentage (8.62 ± 0.208 and 241 ± 72, respectively), where crude fibers percentage varies significantly in the studied groups (*p* < 0.05). Similarly, Group III has rich contents of ash percentage (2.621 ± 0.260) having significant variation, i.e., (*p* < 0.05), while Nitrogen, Magnesium, and potassium percentages were higher in group-III, i.e., 9.179 ± 0.247, 210 ± 22, and 0.816 ± 0.016, respectively. The magnesium, phosphorus, potassium, and carbon contents did not vary significantly in the groups and ranged between 0.18–0.21, 0.14–0.16, 0.72–0.81, and 0.12–0.24, respectively.

### 3.2 Interest correlations among minerals and proximate components

**Figure 5:**
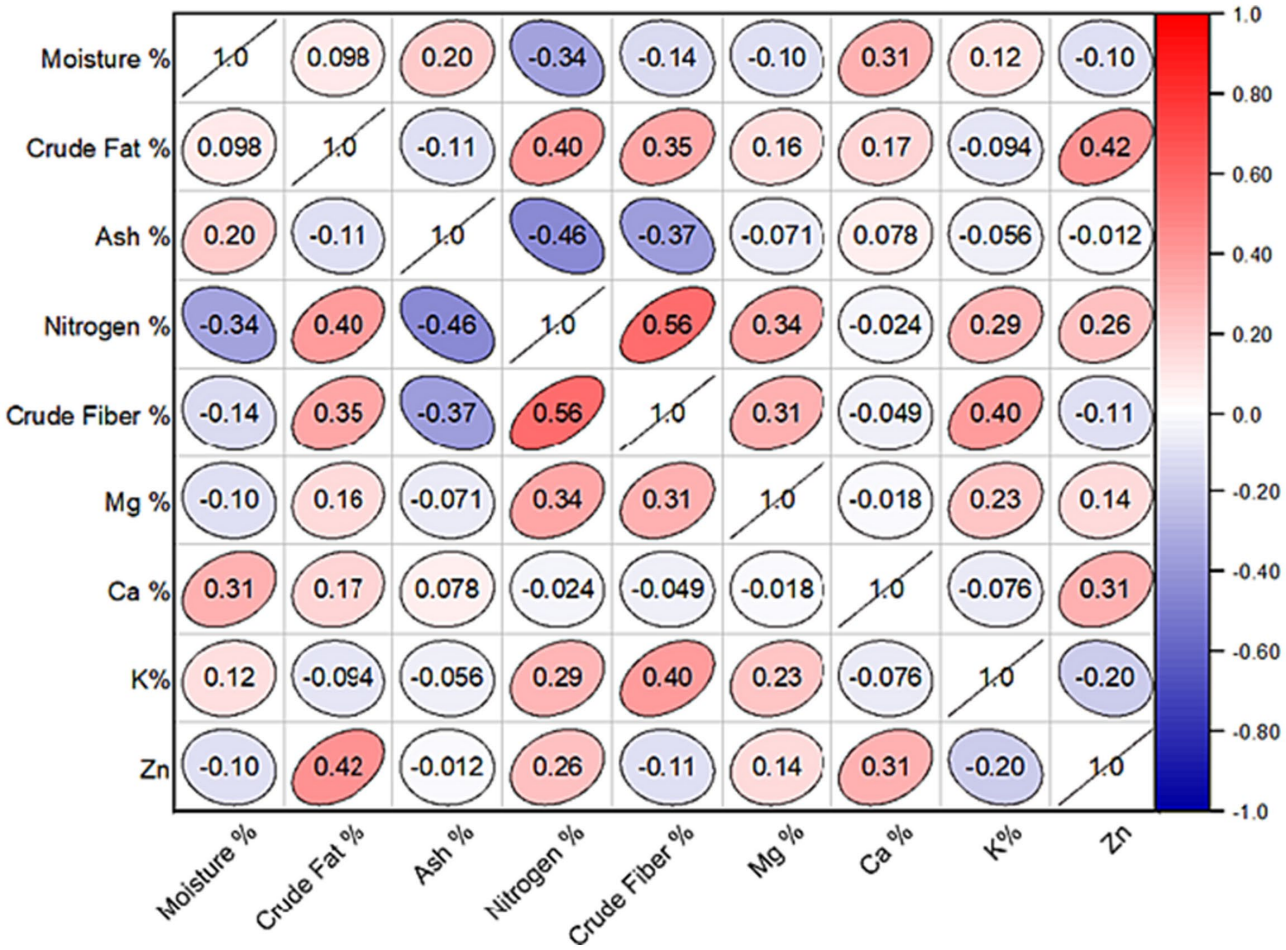
Pearson’s correlation coefficient among the proximate value of 30 different site samples in the study region. Mg, Magnesium; Ca, Calcium; K, Potassium; Zn, Zinc.

The inter-correlation analysis revealed a significant association among the environmental variables recorded from 27 sites. Specifically, the percentage of nitrogen percentage displayed a significant negative correlation with both moisture (*r* = −0.34; *p* < 0.05) and percent ash (*r* = −0.40; *p* < 0.05), respectively. In contrast, there was a significant positive correlation between the percentage of crude fat and the percentages of nitrogen (*r* = 0.40; *p* < 0.05) and crude fiber (*r* = 0.35; *p* < 0.05). Correspondingly, crude fibers had a strong positive correlation with potassium (*r* = 0.40; *p* < 0.05) and nitrogen (*r* = 0.56; *p* < 0.05). These relationships demonstrated the interdependence of mineral and proximate content variables; nevertheless, when examining mineral nutrients, were either non-significant or weak in most cases, as indicated by the correlation coefficients (Figure 5).

### 3.3 Environmental factors associated with *Dryopteris filix-mas* fronds nutrients

The geospatial distribution, topography, soil properties, and the coexistence of plant species richness and diversity are intricately intertwined with *D. filix-mas* populations and the nutrient content of their young fronds displaying great heterogeneity (Table 1). Among the spatial factors, elevation (ranging from 1,822 to 2,207 masl) exhibits significant variation (*F*-value = 6.98, *p* < 0.01) across the nutrient clusters. However, spatial factors like latitudes, longitudes, and aspect degree did not vary significantly. The soil pH in all *D. filix-mas* populations (Cluster-I–III), stemming from a shared parental material of igneous rocks, tends to be somewhat acidic to neutral (pH ≥ 6.99). The pH of soil shows non-significant variation (*F*-value = 0.05, *p* > 0.05), ensuring the availability of soil nutrients to all the plants in the communities. The acidic pH of soils is due to various factors, i.e., parent materials in the form of igneous rock and 12 different kinds of geological materials. The igneous material is because of the area specific position in Hindu-Kush mountain ranges. Nevertheless, phosphorus (7.21–11.58%) and nitrogen (0.02–0.044%) contents show a decrease from Cluster-I to Cluster-III, indicating a clear correlation with altitude (*r* = 0.43; *p* < 0.05, see Appendix Figure 1) and longitude (*r* = 0.26; *p* < 0.05). The three *D. filix-mas* populations exhibit significant differences in soil textures, especially in sand (60–71%, *F*-value = 06.66; *p* < 0.05) and silt (24–36%, *F*-value = 10.23; *p* < 0.01) proportions, which underscore a clear preference for this fern and their minerals-nutrients variability toward acidic to neutral sandy-loam soils. Available phosphorus ranging from 7.21 to 11.58 ppm also varied significantly (*F*-value = 3.65; *p* < 0.05) and notably above the dictation limit (<2.5 mg/kg) as observed in other wild plant populations (e.g., *Dodonea viscosa*). The electrical conductivity ranges from 0.27 to 0.31 μS/cm, exhibiting remarkably low salinity levels in the region, whereas organic matter varies between 0.50 and 0.58%, depending on the specific site and vegetation composition. Likewise, a few soils hydraulic properties, including wilting point (*F*-value 3.76; *p* < 0.05), field capacity (*F*-value =7.79; *p* < 0.001), and available water (*F*-value = 8.33; *p* < 0.001), exhibit statistical variation among the *D. filix-mas* populations (Table 1) but they do not appear to significant impact the frond nutrients characteristics. Furthermore, the first two axes of PCA-ordination (Appendix Figure 2) for environmental variables account for 42.86% of the variance, distinctly associated with various environmental factors that load positively or negatively onto the axes. The first PCA-axis explains 26.80% of the variance and shows a negative correlation with phosphorus and sand while exhibiting positive correlations with clay, silt, wilting point, field capacity, available water and species richness, and diversity. Contrarily, the second PCA-axis (referred to as “PC2”) accounts for 16.06% of the variance and displays positive associations with latitude, pH, electrical conductivity, silt, bulk density, and negative with percent clay, wilting point, and species equitability.

**Table 1:** Environmental and diversity-related variables associated with nutrient parameters in *Dryopteris filix mass*.

Variables	Code	Unit	Groups			*F*	*p* value
			I	II	III		
Latitude	Lat	(°)	34.01 ± 0.4	34.82 ± 0.5	34.80 ± 0.58	0.4	0.68
Longitude	Long	(°)	72.25 ± 0.07	72.24 ± 0.09	72.25 ± 0.09	0.11	0.9
Altitude	Alt	m	2,207 ± 29	1,954 ± 47.2	1,822 ± 154	6.98	0.004
Aspect degree	AD	(°)	246.5 ± 44	208.7 ± 42	217.9 ± 34	0.19	0.83
pH	--	(1:5)	6.98 ± 0.12	6.96 ± 0.098	6.99 ± 0.05	0.05	0.95
Electrical conductivity	EC	S/m	0.27 ± 0.01	0.31 ± 0.03	0.30 ± 0.02	0.35	0.71
Phosphorus	P	(ppm)	11.58 ± 1.6	8.57 ± 1.07	7.21 ± 0.8	3.69	0.04
Nitrogen	N	(%)	0.029 ± 0.03	0.044 ± 0.01	0.02 ± 0.002	0.89	0.42
Potassium	K	(ppm)	89.7 ± 13	96.6 ± 10	111.2 ± 6	1.49	0.24
Organic matter	OM	(%)	0.58 ± 0.07	0.50 ± 0.04	0.51 ± 0.04	0.59	0.56
Clay	--	(%)	4.57 ± 0.8	6.66 ± 0.8	6.14 ± 0.6	1.62	0.22
Silt	--	(%)	24.2 ± 0.8	36.2 ± 3.4	32.42 ± 1.2	6.66	0
Sand	--	(%)	71.1 ± 0.7	60 ± 1.66	61.42 ± 1.7	10.23	0
Wilting point	WP	(%)	0.06 ± 0.0035	0.07 ± 0.003	0.07 ± 0.002	3.76	0.04
Field capacity	FC	(%)	0.16 ± 0.03	0.21 ± 0.01	0.19 ± 0.005	7.79	0
Bulk density	BD	(%)	1.70 ± 0.07	1.58 ± 0.03	1.63 ± 0.02	2.98	0.07
Available water	AW	(%)	0.10 ± 0.01	0.12 ± 0.003	0.11 ± 0.002	8.33	0
Species richness	S	---	9.14 ± 1.43	13.22 ± 0.81	10.57 ± 0.60	4.76	0.02
Shannon-Wiener diversity index	H	---	1.76 ± 0.09	2.06 ± ±0.06	2.00 ± 0.07	3.09	0.06
Evenness or equitability index	J	---	0.83 ± 0.03	0.80 ± 0.018	0.85 ± 0.015	1.89	0.17
Simpson’s index	I/D	---	3.02 ± 0.02	4.03 ± 0.32	3.98 ± 0.21	2.21	0.07

### 3.4 Relationships between influencing factors and Fern fronds nutritional profile

The relationship between nutrient-soil and diversity indices indicated a cumulative variance of 65.8%. Axis 1 played a prominent role, accounting for 45.4% of this variance with a substantial eigenvalue of 3.01, signifying its significant contribution to data distribution (Table 2). Correlation coefficients for the three axes demonstrated the statistical significance of Axis 1 (*r* = 0.88), Axis 2 (*r* = 0.95), and Axis 3 (*r* = 0.74), respectively. Notably, the axes’ eigenvalues were markedly high; Axis 1 retained the highest values (3.01), trailed by Axis 2 (0.94), suggesting a substantial data load on this axis.

**Table 2:** Statistical overview of results (i.e., variance and correlations) extracted from three axes of the Redundancy analysis (RDA) model.

Axis				Axis 1	Axis 2	Axis 3
Eigenvalues				3.01	0.941	0.411
% of variance explained				45.4	14.2	6.2
Cumulative % explained				45.4	59.6	65.8
Pearson Corr., Response-Pred.*				0.881	0.951	0.744
Kendall Corr., Response-Pred.				0.608	0.801	0.543
Variables	Correlation			Biplot Scores		
	Axis 1	Axis 2	Axis 3	Axis 1	Axis 2	Axis 3
Latitude	0.047	−0.42	0.151	0.049	−0.243	0.058
Longitude	−0.095	0.15	−0.216	−0.099	0.087	−0.083
Altitude	0.228	0.137	−0.083	0.238	0.08	−0.032
Aspect degree	−0.163	−0.069	0.353	−0.17	−0.04	0.136
pH	0.32	0.18	−0.057	0.078	0.105	−0.022
Electrical conductivity	−0.097	−0.059	−0.305	−0.101	−0.034	−0.117
Phosphorus	−0.21	0.317	−0.033	−0.219	0.185	−0.013
Nitrogen	−0.146	−0.143	0.291	−0.152	−0.083	0.112
Potassium	0.437	−0.44	−0.091	0.455	−0.254	−0.035
Organic matter	0.068	0.17	0.031	0.071	0.099	0.012
Clay	−0.056	−0.21	0.041	−0.058	−0.122	0.016
Silt	−0.139	−0.17	−0.043	−0.145	−0.099	−0.016
Sand	−0.039	0.253	0.086	−0.04	0.147	0.033
Wilting point	−0.04	−0.27	0.002	−0.042	−0.159	0.001
Field capacity	−0.15	−0.24	0.265	−0.156	−0.141	0.102
Bulk density	0.11	0.216	−0.259	0.114	0.125	−0.1
Available water	0.068	−0.21	−0.168	0.07	−0.122	−0.064
Species richness	−0.036	−0.32	−0.249	−0.038	−0.186	−0.096
Shannon-Wiener diversity index	0.318	−0.22	0.035	0.331	−0.13	0.014
Evenness or equitability index	0.493	0.163	0.401	0.513	0.095	0.154
Simpson’s index	0.525	−0.017	0.255	0.547	−0.01	0.098

**Figure 6:**
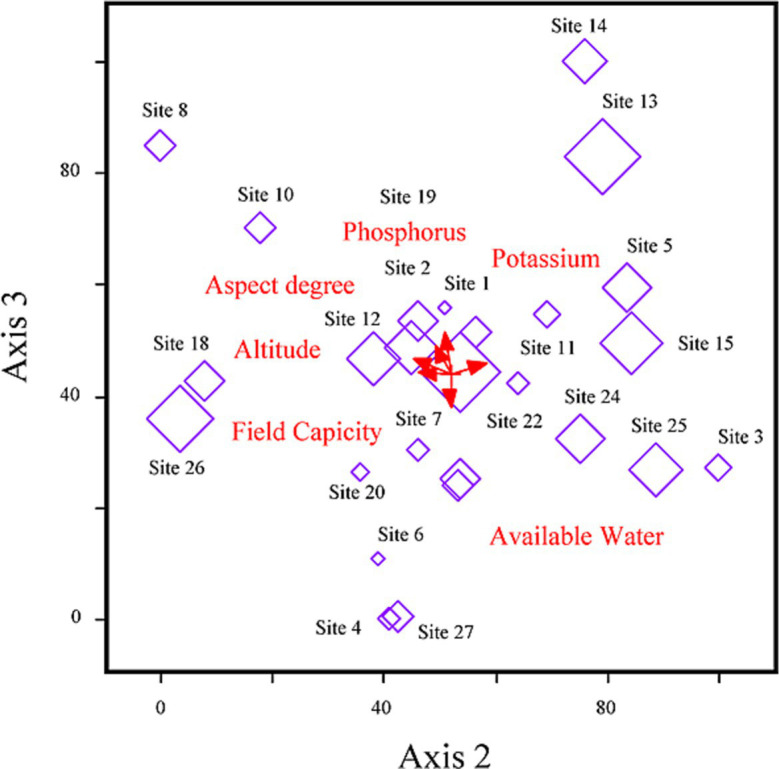
RDA-biplot model exhibiting relationships between environmental and plant species coexistence, fern fronds minerals, and nutritional profile.

The nutrient composition of *D. filix-mas* and assemblages were primarily shaped and influenced by topography and soil gradients (see Figure 4). The distribution and abundance of nutrients were notably affected by 10 indicator parameters among the 20 environmental, soil, and coexistence plant species diversity variables examined. Among these influencing factors, aspect degree (*r* = 0.35), pH (*r* = 0.32), potassium (*r* = 0.43), wilting point (*r* = 0.27), available water (*r* = 0.21), species richness (*r* = 0.32), Evenness index (*r* = 0.49), and Shannon-winner index (*r* = 0.36), displayed statistically significant positive correlations with the first ordination axis. Conversely, the axis is the elevation gradient axis having (*r* = −0.41), potassium (*r* = −0.43), and Shannon-wiener diversity index (*r* = −0.22), respectively, while sand % (*r* = 0.25) revealed a positive correlation. Axis 2 accounted for 6.8% of the fern fronds minerals and nutritional variability and demonstrated a negative relationship with field capacity (*r* = −0.24), wilting point (*r* = −0.27), nevertheless exhibiting positive correlations with phosphorus (*r* = 0.31), and bulk density (*r* = 0.21) thereby providing a favorable environment for fern growth and its nutrients retention capacity. Although, species and stands showed some dispersion clusters along axes 1 and 2, the RDA-ordination biplot did not effectively distinguish the nutrient groups (Figure 5). However, the model effectively underscored the significance of key environmental factors with biplot scores of −0.29 for phosphorus, 0.45 for potassium on axes 1, and elevation (−0.24) on axis 2, indicating its high significance (Figure 6).

These results can be summarized as follows:

- *Dryopteris filix-mas* has significant quantities of vital nutrients, i.e., moisture, crude fibers, ash, magnesium, calcium, potassium, and zinc.
- Various environmental and soil variables associated with *D. filix-mas* show significant variation, indicating its role in maintaining the nutrient contents.
- The PCA analysis revealed the role of environmental, soil, and diversity variables on nutrient concentration as environmental variables > soil variables > diversity indices.
- The nutrients-environment relationship was assessed using RDA analysis, which shows the promising role of altitude, aspect degree, field capacity, available water, potassium, and phosphorus contents of soil in shaping the nutrient concentrations of *D. filix-mas*.

## 4 Discussion

The nutritional value, chemical composition, and effects of soil features and diversity indices on nutrient contents were determined using a proximate analysis of the *D. filix-mas*, used as a wild edible vegetable. The highest moisture content was found in Group II plants, followed by Group III and I. The moisture contents are critical for sustaining vegetables’ flavor and shelf life and for the vegetable cells to remain turgid and fresh (54). However, the moisture content measured in this study was typically lower than the moisture value measured by Martins et al. (55). Due to this reason, the vegetable has less life and is usually sold as fresh in the market. The vegetables were high in crude protein and fiber %, indicating that this vegetable is a good source of protein. Wild plants are an excellent source of protein in most rural and underdeveloped regions of Pakistan (56). Similarly, Mih et al. (57) and Sundriyal and Sundriyal (58) also reported that wild edible vegetables can be used as a protein source and has also been confirmed by Ullah et al. (45) in *P. plebejum* having the highest crude fiber content (12.74%). These findings revealed that this vegetable might be used in diet to supplement protein and fiber deficiencies in low-income households.

Similarly, the fiber contents ranged from 6.6 to 8.45% within the nutrient groups. The carbohydrates the body cannot process, adding bulk to the diet, are critical for appropriate digestive system functions (59). Our findings were consistent with Rehman and Adnan (60) and Muchuweti et al. (61) because they recorded the same crude fiber levels as the present investigation. Similarly, the species was found to have a rich source of minerals, e.g., Mg (0.20–021%), Ca (0.14–0.16%), K (0.72–0.82%), and Zn (0.12–0.24 mg/Kg). Mg is required in plasma and extracellular fluid to sustain osmotic balance (62). The findings reveal that wild vegetables include significant levels of Ca, K, and Zn, which are necessary nutrients for living organisms and are needed for the synthesis of chlorophyll and cell walls, as well as the transport of oxygen and electrons (63). According to the current findings, wild foods are an excellent source of K. Zn is a necessary trace element for protein synthesis, nucleic acid synthesis, and proper body development (64), and the obtained value agrees with Trichopoulou et al. (65). The result was consistent with Imran et al. (66). Therefore, the rural population’s intake of these species is important for medicine and nutrition. The findings revealed that most wild edible vegetables had significant nutritional value and should be included in the daily diet of rural people. This fantastic vegetable is high in minerals, crude fat, crude protein, and crude fiber and is useful for treating various ailments.

Studied site spatial factors, soil characteristics, and diversity indices variations observed could explain the response in the proximal composition of the evaluated species, where the sites showed neutral pH. According to de Albuquerque et al. (67) and Misra and Tyler (68), the pH and nutrient availability at these sites were directly controlled by the soil texture and water availability. The neutral soil pH additionally the availability and uptake of nutrients from the soil, that aid in increasing nutritional value of a plant species (69). However, the decrease in pH may negatively interfere the nutrient availability which is not the case in our study indicating the soil condition provide suitable condition for availability and uptake of nutrients. Moreover, the soil’s primary nutrient availability was due to the pH’s neutrality and greater levels of organic matter, which also improved the availability of nutrients. As previously reported in fertilizer applications in various studies (70–72). The abundance of organic matter and micronutrients in soils positively influenced a higher accumulation of proximate nutrients, and the higher nutrient availability in soils promoted better nutritional quality in storage roots (73). The optimal level of nutrient in fertilizers enhances the accumulation of nutrients in different parts of a plant (74). For example, addition of nitrogen fertilizer increases the amount of phenolics, triggering flavonoids pathway enzymes (75). However, the nutritional value of this species in its native environments has, nevertheless, been noted. Therefore, evaluating its nutritional value in these habitats is vital for overcoming the food scarcity in future for nation living in under-developed conditions. However, weather circumstances such as seasonal rainfall and temperature may have directly impacted crop growth and nutrients availability (76), phytosanitary measures, germination, and harvesting time (77). These weather circumstance and soil characteristics are primarily linked to environmental conditions that can influence the quality, in particular nutritional content and profile (78). Different responses to seasonal variations were reported in several crops, such as broccoli, kale, and turnip (79), however, no such valuable data are available regarding the fern species. Soil and climate may be more important than believed and warrant further evaluation alongside the commonly implicated factors and anthropogenic disturbance as suggested in studies conducted by Ullah et al. (38).

The study demonstrates that the species is a significant source of nutrients comparable to those found in more widely consumed and domesticated vegetable plants. The comparison with previous literature has been presented in Table 3. In addition, the species also have a comparable quantity of nutrients when compared with exotic fruit trees, including the apple, avocado, guava, jackfruit, papaya, pineapple, and mango. For instance, for crude nitrogen, crude lipids, crude ash, and crude fiber, the proximate contents of exotic species (Avocado, Mango, and Paya) and study species [*D. filix-mas*] vary from 0.5 to 6.0 [7.1–9.1], 0.1 to 1.54 [5.8–8.08], 0.5 to 1.6 [2.0–2.62], and 0.7 to 6.8 [6.61–8.62], respectively. This shows that WEPs could improve the security of food and nutrition. Especially for underdeveloped countries where daily fruit intake is much lower than the 200 g per person daily recommendation (83). In support of this, our research revealed that the studied species could provide around 10–15, 39–72, 17–38, and 2–7%, respectively, of the daily recommendations for calcium, potassium, magnesium, and zinc (Figure 2). In many communities, WEVs may also play a key role in sustaining family nutrition, particularly during lean seasons, periods of poor agricultural productivity, and droughts brought on by climate change (83). The findings of this research further support the significance of conserving and using these food-producing species sustainably to ensure their continued contribution as a dependable source of food, nutrition, and medicine for those who rely on sustainable agricultural systems.

**Table 3:** Comparison of *Dryopteris filix-mas* nutrients contents with widely used flowering vegetables of the area.

Plant name	DF.	TP.	RP.	BR.	UD.	CE.	AS.	*CA.*
Moisture %	1.64	8.01	13.6	11.5	67.19	62.44	77.8	75.33
Crude Fat %	6.68	2.03	0.89	0.22	2.58	2.64	3.34	2.64
Ash %	2.23	34.8	0.79	0.63	3.07	2.77	2.85	2.37
Nitrogen %	8.19	9.19	0.88	1.98	6.68	10.66	4.53	10.66
Crude Fiber %	7.64	43	0.67	0.75	15.38	17.47	7.39	17.47
Mg %	0.20	***	17	13.2	0.06	0.01	0.078	3.11
Ca %	0.16	***	***	***	0.153	0.306	0.175	1.084
K%	0.77	5.1	***	***	***	***	***	***
Zn (mg/Kg)	17.88	0.2	***	***	0.41	0.78	0.47	0.14
Citation	Current	(80)	(81)		(82)			

D.F., *Dryopteris filix-mas*; T.P., *Trianthema portulacastrum*; R.P., *Raphanus sativus*; B.R., *Braissuca rapa*; U.D., *Urtica dioica*; C.E., *Caralluma edulis*; A.S., *Amaranthus spinosis*; C.A., *Chenopodium album*.

## 5 Conclusion and recommendations

According to preliminary studies, *D. filix-mas* have good nutritional and mineral value. The essential nutrients and minerals Ca, Mg, and K, as well as crude fibers and protein, are present in adequate amounts to suit human dietary needs. Traditional wild veggies are very inexpensive and readily available to rural communities. According to this research, wild vegetables constitute a good source of nourishment. We additionally contend that further investigation into chemical and mineral concentrations, as well as testing it in various ways using substantial amounts of samples collected in different regions of the country, should be accomplished to choose the more preferred, lucrative, and flexible to broader ecoclimatic conditions for potential domestication and scaling up of various agroforestry systems. Finally, the following recommendations are suggested based on the knowledge gained from this study and the literature analysis.

- More investigation on antinutrient substances, heavy metal concentrations, and vitamins will be beneficial for evaluating their nutritional safety.
- To encourage their contribution to household diet, socioeconomic role, and environmental protection, more research is needed on postharvest storage, food value and consumption, and commercialization of WEVs per household. This is in addition to documenting the nutritional contents of WEVs.
- Improve farmers’ knowledge of significant indigenous plant species via nutritional messaging communication to increase public awareness of WEVs’ potential to improve diets and nutrition.
- Promote domestication and development efforts for these species by choosing superior varieties based on important characteristics like plant size and nutritional value.

WEVs conservation using a more generally planted, organically managed, and maintained environment for sustenance and possibly financial benefits.

## Data availability statement

The original contributions presented in the study are included in the article/Supplementary material; further inquiries can be directed to the corresponding author.

## Author contributions

NK: Conceptualization, Data curation, Formal analysis, Software, Writing – original draft. RU: Data curation, Formal analysis, Software, Writing – original draft. MO: Funding acquisition, Project administration, Resources, Supervision, Writing – review & editing. MA-M: Formal analysis, Resources, Validation, Visualization, Writing – review & editing. IS: Formal analysis, Methodology, Resources, Writing – review & editing. HA-H: Formal analysis, Methodology, Resources, Writing – review & editing. TA: Formal analysis, Methodology, Software, Writing – review & editing. HA: Formal analysis, Methodology, Supervision, Writing – review & editing.
