# Supplementary material for: Ecological factors affecting minerals and nutritional quality of “Dryopteris filix-mas (L.) Schott”: an underutilized wild leafy vegetable in rural communities
Source: Front Nutr. 2024 Feb 20;11:1276307. doi: 10.3389/fnut.2024.1276307 (PMC10916005; doi:10.3389/fnut.2024.1276307)
Supplement: Supplementary file 1 [file Data_Sheet_1.docx]

Appendix Table 1: Proximate and micronutrients concentration of *Dryopteris Filix-mas*

| Variables | Group 1 | Group 2 | Group 3 | F | P-value |
| --- | --- | --- | --- | --- | --- |
| Moisture % | 1.26±0.221 | 1.98±0.334 | 1.66±0.123 | 2.68 | 0.09 |
| Crude Fat % | 6.50±0.23 | 8.097±0.122 | 5.838±0.352 | 17.02 | 0.00 |
| Ash % | 1.59±0.172 | 2.02±0.22 | 2.621±0.260 | 4.72 | 0.02 |
| Nitrogen % | 9.179±0.247 | 8.804±0.302 | 7.141±0.349 | 13.47 | 0.00 |
| Crude Fiber % | 8.45±0.186 | 8.62±0.208 | 6.615±0.369 | 14.01 | 0.00 |
| Magnesium (%) | 0.21±0.022 | 0.189±0.019 | 0.20±0.025 | 0.10 | 0.90 |
| Calcium (%) | 0.147±0.013 | 0.161±0.010 | 0.161±0.011 | 0.42 | 0.66 |
| Potassium (%) | 0.816±0.016 | 0.78±0.047 | 0.72±0.052 | 0.88 | 0.42 |
| Zinc (mg/Kg) | 12.3±0.76 | 13±0.42 | 24.1±0.72 | 1.11 | 0.34 |


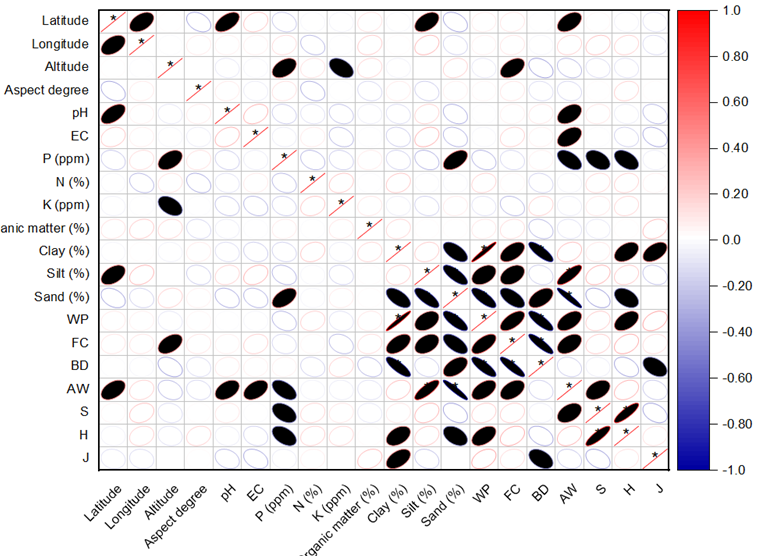


Appendix Figure 1. Correlation of the geospatial, topographic, soil physiochemical and species-diversity variables affecting the nutrient contents of *D*. *filix-mas*.

Note: Figure legends are same as that of table 1

Appendix Table 2: Principal Component analysis axis loading of the spatial and soil variables and diversity indices

| PCA number | Eigenvalue | PC 1 | PC 2 | PC 1 | PC 2 | Variables |
| --- | --- | --- | --- | --- | --- | --- |
|  |  | 26.80% | 16.06% | 26.80% | 16.06% |  |
| 1 | 5.4 | 0 | 0 | 0.15 | 0.29 | Latitude |
| 2 | 3.2 | 0 | 0 | 0.10 | 0.13 | Longitude |
| 3 | 2.4 | 0 | 0 | 0.02 | 0.18 | Altitude |
| 4 | 1.6 | 0 | 0 | 0.01 | 0.12 | Aspect degree |
| 5 | 1.5 | 0 | 0 | 0.09 | 0.31 | pH |
| 6 | 1.4 | 0 | 0 | 0.082 | 0.28 | EC |
| 7 | 0.9 | 0 | 0 | 0.22 | 0.14 | P (ppm) |
| 8 | 0.8 | 0 | 0 | 0.07 | 0.10 | N (%) |
| 9 | 0.7 | 0 | 0 | 0.05 | 0.00 | K (ppm) |
| 10 | 0.6 | 0 | 0 | 0.044 | 0.15 | Organic matter (%) |
| 11 | 0.5 | 0 | 0 | 0.31 | 0.32 | Clay (%) |
| 12 | 0.4 | 0 | 0 | 0.29 | 0.24 | Silt (%) |
| 13 | 0.3 | 0 | 0 | 0.40 | 0.12 | Sand (%) |
| 14 | 0.2 | 0 | 0 | 0.37 | -0.2 | WP (%) |
| 15 | 0.1 | 0 | 0 | 0.32 | 0.16 | FC (%) |
| 16 | 0.0 | 0 | 0 | 0.30 | 0.348 | BD (%) |
| 17 | 0.0 | 0 | 0 | 0.34 | 0.29 | AW (%) |
| 18 | 0.009 | 0 | 0 | 0.21 | 0.14 | S |
| 19 | 0.0 | 0 | 0 | 0.26 | 0.08 | H |
| 20 | 0.0 | 0 | 0 | 0.05 | 0.39 | J |

Note: PCA (Principal component axis); PC (Principal component): the rest of the legends are same as that of table 1


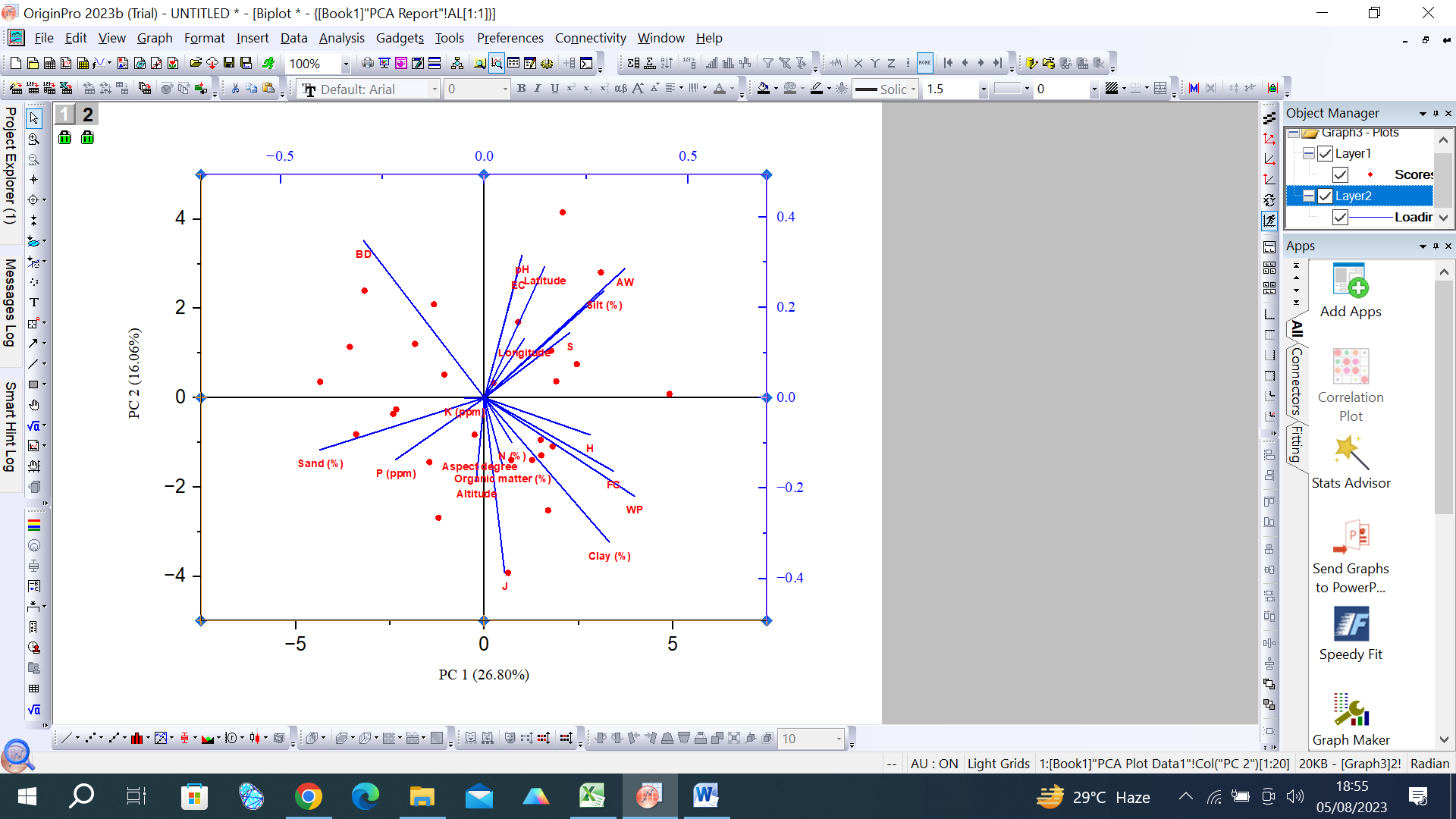


Appendix Figure 2: PCA biplot of the spatial and soil variable and diversity indices

Note: Figure legends are same as that of Table
